# Cell-to-cell lactate shuttle operates in heart and is important in age-related heart failure

**DOI:** 10.18632/aging.102818

**Published:** 2020-02-08

**Authors:** Agnieszka Gizak, James A. McCubrey, Dariusz Rakus

**Affiliations:** 1 Department of Molecular Physiology and Neurobiology, University of Wrocław, Wrocław 50-137, Poland; 2 Department of Microbiology and Immunology, Brody School of Medicine at East Carolina University, Greenville, NC 27858, USA

**Keywords:** cardiac maturation, lactate shuttle, FBP2, HIF, glycolysis

## Abstract

Recent studies have revealed a resemblance of a HIF-regulated heart and brain glycolytic profiles prompting the hypothesis that the classical cell-to-cell lactate shuttle observed between astrocytes and neurons operates also in heart – between cardiac fibroblasts and cardiomyocytes. Here, we demonstrate that co-culturing of cardiomyocytes with cardiac fibroblasts leads to orchestrated changes in expression and/or localization pattern of glucose metabolism enzymes and lactate transport proteins in both cell types. These changes are regulated by paracrine signaling using microvesicle-packed and soluble factors released to the culture medium and, taken together, they concur with the cardiac lactate shuttle hypothesis. The results presented here show that similarity of heart and brain proteomes demonstrated earlier extend to physiological level and provide a theoretical rationale for designing novel therapeutic strategies for treatment of cardiomyopathies resulting from disruption of the maturation of cardiac metabolic pathways, and of heart failure associated with metabolic complications and age-related heart failure linked with extracellular matrix deposition and hypoxia.

## INTRODUCTION

During the last twenty years several lines of evidence have accumulated, based mainly on observations of cell co-culture models, that cells building some organs (neurons and astrocytes in brain, cancer cells and cancer associated fibroblasts in some cancers) communicate with each other exchanging energetic substrates such as lactate and glutamine (for review see: [1, 2]), and releasing molecules which affect physiology and morphology of their in vivo partner cells, for example, significantly altering expression of metabolic enzymes [e.g. 3].

Our recent proteomic studies of mouse organs have revealed that the expression pattern of energy metabolism enzymes in mouse heart closely resembles mouse brain [4]. Brain and heart are built of two major types of cells: neurons and astrocytes, and cardiomyocytes and fibroblasts, respectively. It has been shown that both neurons and cardiomyocytes preferentially utilize lactate, even in the presence of glucose, which makes them highly sensitive to hypoxia [5]. It has also been shown that astrocytes take up the majority of the brain glucose and metabolize it to lactate which is then transported to neurons and enters the Krebs cycle [1]. However, up to now, practically none of studies on the expression/activity of proteins in the whole heart assumed that the fibroblast-cardiomyocyte cross-talk may significantly influence these parameters.

Therefore, considering the resemblance of the heart and brain glycolytic profiles, and physiological response of neurons and cardiomyocytes to hypoxia, we have proposed that the classical cell-to-cell lactate shuttle operates also in heart where fibroblasts deliver lactate to cardiomyocytes [4, 6].

Although our hypothesis has been backed up with predominantly fibroblastic localization of two proteins responsible for a high basal glucose uptake (hexokinase 1) and release of lactate from a cell (monocarboxylate transporter 4, MCT4) [6], it relied mostly on results of proteomic studies of the whole rodent heart. Thus, we decided to test if our hypothesis has any relevance to intercellular relations in heart, i.e. if cardiac fibroblasts can influence cardiomyocytic metabolism just as they do in cancer, or as astrocytes influence neuronal processes. To this end, we cultured for 48 h mouse cardiac myocytes (HL-1 cell line) alone or together with fibroblasts isolated from mouse heart. Then, we checked localization of proteins involved in regulation of glucose metabolism and proliferation, and searched for the possible mechanism by which the cells may communicate and mutually modify their biology. Results of our experiments demonstrate that co-culturing of cardiomyocytes with fibroblasts leads to orchestrated changes in metabolic protein expression/localization which concur with the fibroblasts-to-cardiomyocytes lactate shuttle hypothesis, and that these changes are regulated both by microvesicle-delivered and soluble factors of the culture medium. Moreover, the similarity of aging-related changes in brain and heart might suggest that the metabolic cross-talk between fibroblasts and cardiomyocytes is impaired in old animals and also in animals suffering from obesity-related diabetic complications.

## RESULTS AND DISCUSSION

**Figure 1:**
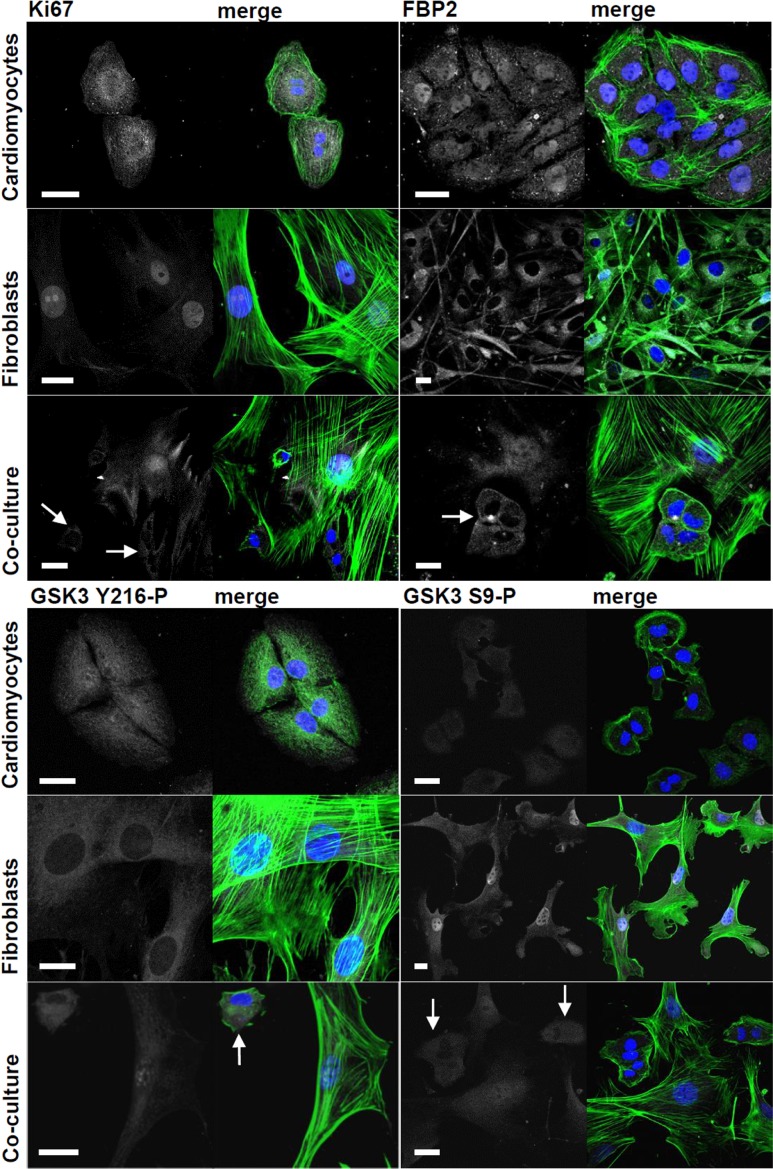
**Changes in subcellular localization and immunostaining intensity of Ki-67, FBP2 and FBP2-regulating kinase GSK3 evoked by cardiomyocytes-fibroblasts co culture.** On merged pictures actin appears as green and nuclei are shown in blue. White arrows point to cardiomyocytes. Bar = 20 μm.

The most pronounced manifestation of the cardiomyocyte-fibroblast cross-talk was reduction of the proliferative capacity of both cell types assessed by cellular expression of Ki-67, a protein widely accepted as a proliferation marker. In the monocultures, over 90% of both cell types had Ki-67–positive nuclei (Figure 1; Supplementary Figure 1A). However, co-culture of these cells significantly reduced the number of Ki-67–positive nuclei of cardiomyocytes (almost 2-fold) and, much less markedly (~1.3x), fibroblasts (Figure 1; Supplementary Figure 1A).

To verify this result the cell cycle analysis was performed. 48h co-culture of cardiomyocytes and fibroblasts led to over 1.2-fold increase of percentage of cells in G0/G1 phase (from over 52% in the monocultures to 67% in the co-culture), and to about 1.8-fold decrease of percentage of the cells in G2/M phase (from over 30% in both monocultures to ~17% in the co-cultures) (Supplementary Figure 1B).

This may indicate a transition of the co-cultured cells from hyperplasia to hypertrophy which mimics the postnatal shift in mode of cardiac growth [7], and should coincide with decrease of glycolytic potential of cardiomyocytes (switching from the “fetal” to “mature” phenotype) [8].

Therefore we tested the subcellular localization of fructose 1,6-bisphosphatase 2 (FBP2, so-called muscle isozyme), a moonlighting protein which, aside from regulation of glycogen synthesis from carbohydrate precursors, is involved in cell cycle-dependent events [9] and protection of cells against stress conditions [10, 11].

It has been demonstrated that nuclear accumulation of the enzyme is related to proliferative capacity of muscle cells [12], and that in HL-1 cardiomyocytes, it oscillates in a cell cycle-dependent manner [9] and is also dependent on GSK3 activity [10].

In HL-1 cells monoculture, FBP2 had nucleo-cytoplasmic localization [13] (Figure 1). In contrast, in cardiac fibroblasts monoculture, majority of the cells lacked substantial nuclear immunostaining and FBP2 seemed to be distributed homogeneously in cytoplasm. One of the most conspicuous effects of cardiac myocytes-fibroblasts co-culture was disappearance of FBP2 from nuclei of cardiomyocytes and accumulation of the protein in nuclei of fibroblasts (Figure 1).

Thus, to check what triggered the nucleo-cytoplasmic shuttle of FBP2 we investigated the changes in GSK3 phosphorylation level.

In the co-culture, there were no observable changes in the intensity of immunostaining against total form of GSK3 (data not shown) in any of the two cell types. However, a slight (1.4x) decrease of the active form of GSK3 (Y216-P) and similar (1.5x) increase of the inactive form (S9-P) was observed in cardiomyocytic nuclei (Figure 1; Supplementary Figure 1D). On the other hand, in the fibroblasts’ nuclei, the amount of the active form of GSK3 increased (2.3x) and inactive form (S9-P) decreased (2.5x) (Supplementary Figure 1D).

GSK3 is a multifunctional kinase and its inhibition has an anti-hypertrophic effect on heart and reduces FBP2 nuclear retention [10, 14]. Since both in cardiomyocytes and in fibroblasts, the observed changes of phosphorylation status of the kinase were limited to nuclear GSK3 they were probably related to other functions of GSK3 than regulation of glycogen synthesis (e.g. regulation of FBP2 nucleo-cytoplasmic shuttle).

Thus, taking into account all the above results, it can be inferred that co-culture influences the cell cycle progression in both cell types, and reduces proliferative potential of cardiac myocytes – a phenomenon associated with postnatal maturation that culminates in a decreased cardiomyocyte number in aging hearts.

**Figure 2:**
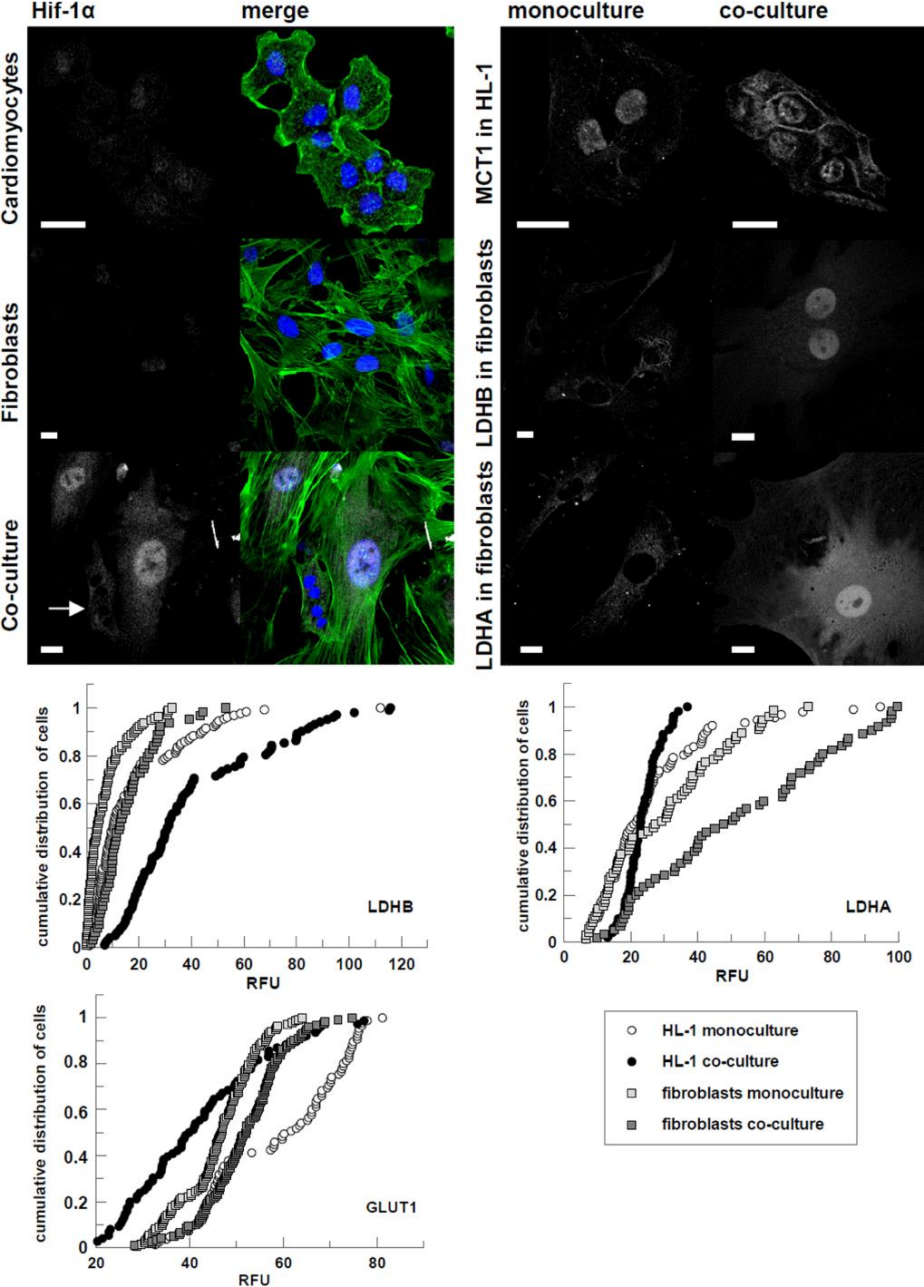
**Changes in subcellular localization and immunostaining intensity of HIF-1α and proteins involved in glucose transport, and lactate transport and production evoked by cardiomyocytes-fibroblasts co culture.** On merged pictures actin appears green and nuclei are shown in blue. White arrows point to cardiomyocytes. Bar = 20 μm. If the changes in fluorescence subcellular distribution were not evident, corrected total cell fluorescence of individual cells was calculated and presented in percentage frequency graphs. RFU – relative fluorescence units.

Concomitantly with the FBP2 distribution changes seen in the co-culture, a noticeable (4-fold, p<0.001) increase of anti-HIF-1α (Hypoxia-inducible factor 1α) staining was observed in fibroblasts, especially in the nuclei (Figure 2; Supplementary Figure 1C). This was in contrast to the hypothesized role of FBP isoforms in cancers. It has been shown that in renal carcinoma, the liver isozyme (FBP1) decreases the rate of glycolysis exerting an antagonistic effect both on the rate of glycolysis and on the level of HIF [15]. It has been also demonstrated that FBP2 may suppress glycolytic pathway in gastric cancer [16]. Contrarily, it has been shown that HIF1α can increase FBP transcript about five-fold during 48 h of hypoxia [17]. However, since in our study, the cardiomyocyte-fibroblast co-culture was kept in normoxic conditions thus, the observed changes could not be explained by the effect of low oxygen availability. Nevertheless, since the alteration FBP2-related fluorescent signal intensity was confined to nuclear compartment the observed changes are most probably related to a moonlighting function of the enzyme which is not directly related to its catalytic action.

HIF is the Nobel Prize-worthy main regulator of cellular metabolic response to hypoxia [18] and it stimulates the expression of practically all glycolytic enzymes in healthy and cancer cells [19, 20]. However, deletion of HIF-1 in cardiomyocytes affects heart function even under normoxic conditions [21]. Expression of HIF-1α can be induced by growth factors, and at the protein level HIF-1α is stabilized by products of glycolysis, especially pyruvate, which creates a feed-forward signaling mechanism [22]. Importantly, this stabilization is independent on hypoxia [23].

Conditions wherein cells express hypoxia proteins regardless of the oxygen status are described as “pseudohypoxia”. It parallels to Warburg effect in which HIF-1 up-regulates genes related to glycolytic energy metabolism in normoxia and which is thus defined as aerobic glycolysis. Both the states are most frequently mentioned in relation to cancer cells. The pseudohypoxic state is often observed at the tumor–stromal interface. However, the acquisition of pseudohypoxic phenotype by cells has been observed in numerous physiological as well as pathological conditions, including diabetes, inflammation, differentiation and aging [24–27]. Pseudohypoxic and Warburg effect displaying cells are lactic acid producing due to HIF-1α-induced up-regulation of genes related to glycolysis and also to glucose and lactate transport [28, 29].

Moreover, it has been shown that stabilization of HIF-1α (not only during stress but also at baseline conditions) can trigger combined adaptations in glutamine and glucose metabolism: stimulating glutathione synthesis from glutamine and “sparing” glucose for maintenance of energy status of cells. In such cells, an increased 13C incorporation into glycolytic intermediates (i.e. lactate) and decreased incorporation into TCA cycle intermediates were observed [30]. This observation is in agreement with papers demonstrating that HIF-1 can induce changes leading to inhibition of the PDH complex [31, 32] what in turn, reduces entry of glucose-derived carbon into the TCA cycle increasing conversion of pyruvate to lactate. However, such changes leading to the lactate-producing (Warburg-like) phenotype has never been reported previously in cardiac fibroblasts.

Consistently with the alterations expected in pseudohypoxic/Warburg effect displaying cells, we observed that the co-culturing increased glucose transporter 1 (GLUT1)-related fluorescence in fibroblasts (D=0.29, p<0.001), at the same time reducing it in HL-1 cells (D=0.45, p<0.001) (Figure 2). In turn, fluorescence related to the B isoform of lactate dehydrogenase (LDHB) served to convert lactate to pyruvate [33] increased in cardiomyocytes (D=0.54, p<0.001). It also increased slightly in fibroblasts, but only in nuclei (D=0.47, p<0.001) (Figure 2), which suggests that it is related to a transcription regulator role of LDH [34]. We also observed migration of monocarboxylate transporter 1 (MCT1), the isoform facilitating L-lactate influx, to membrane of cardiomyocytes in co-culture (Figure 2). It is in accordance with our previous proteomic studies showing that in mouse heart, the high amount of enzymes involved in lactate uptake and its oxidation to pyruvate reflects the phenomenon of lactate consumption by this organ [6].

Although we have also shown that in mouse heart, the MCT4 transporter, primarily expressed in highly glycolytic cells and used to facilitate lactic acid efflux, is present mainly in fibroblasts, we found no observable differences of MCT4-related fluorescence in fibroblast between mono- and co-cultures (data not shown) which suggests that fibroblasts express this transporter independently on culture conditions.

On the other hand, the expression of lactate dehydrogenase A (LDHA), the isoform suited to pyruvate-to-lactate conversion [33], was increased in fibroblasts co-cultured with cardiomyocytes (D=0.39, p<0.001). Changes in cardiomyocytes from co-cultures were less unambiguous (p=0.03). Moreover, just like LDHB, the enzyme showed the tendency to accumulate in nuclei. This accumulation, however, might be related to moonlighting functions of LDHA. The enzyme has been shown to participate in a regulation of gene expression by participation in the Oct-1 co-activator S (OCA-S) transcription complex formation [34] and by controlling availability of NAD^+^ for the sirtuin-1 deacetylase system [35]. It has been also demonstrated that LDHA up-regulates HIF-1α expression in normoxic conditions by enhancing lactate production (see in [33]).

In search for support of our hypothesis, we tested if co-culturing of cardiomyocytes with fibroblasts can influence the regulatory enzymes of glycolysis (HK, PFK, PK), the main regulator of stability of glycolytic complex in a cell (PGAM), and aldolase – the enzyme occupying central position in glycolysis and gluconeogenesis.

**Figure 3:**
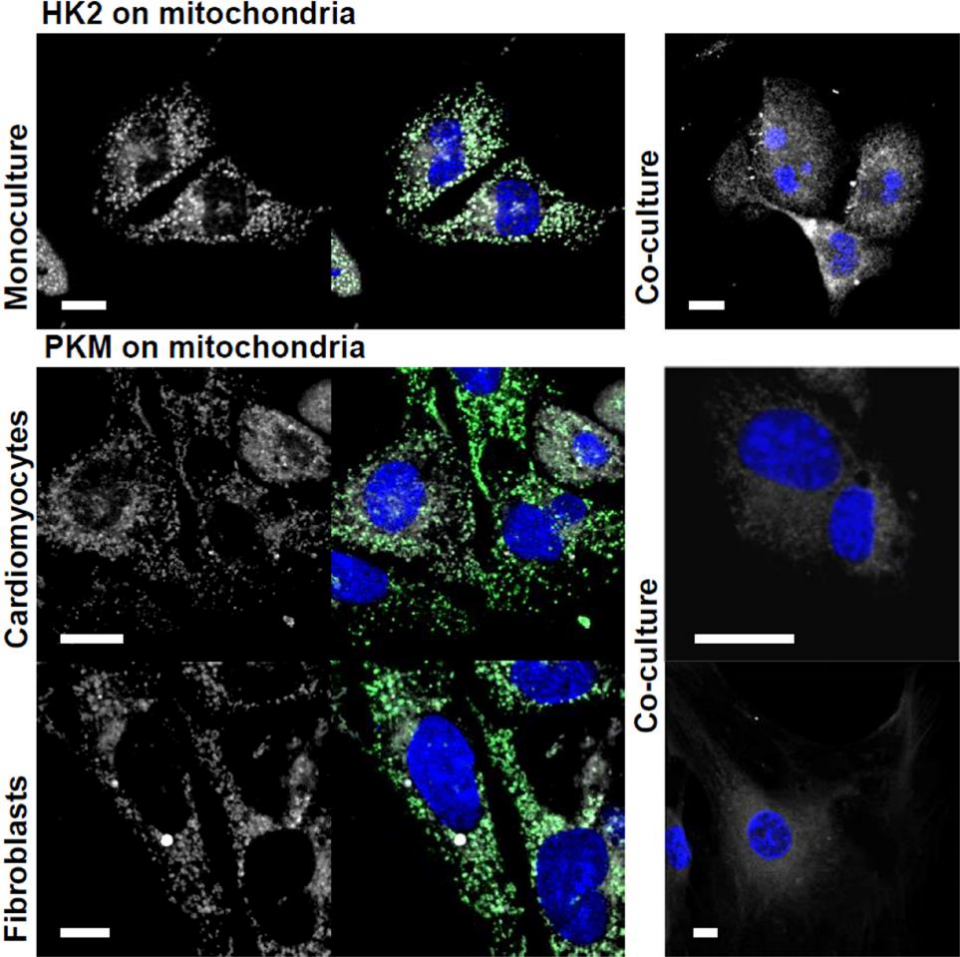
**Co-culturing-induced changes in mitochondrial localization of HK2 and PKM.** The studied proteins are shown in white, mitochondria – in green and nuclei – blue. Bar = 10 μm.

Our previous study has demonstrated that hexokinase 1 (HK1), the isoform typical of high glucose uptake cells, is predominantly localized in cardiac fibroblasts [6]. Here, we show that HK2, the main isoform of cardiac myocytes, is localized on mitochondria of HL-1 cells in the monoculture, but not when their were co-cultured with fibroblasts (Figure 3). This may reflect an adaptation of cardiomyocytes to co-culture-induced changes in metabolic conditions (i.e. the increased supply of lactate) and result in the redirection of glucose-6-phosphate (G6P) from glycolysis to new metabolic routes in cardiomyocytes. It has been shown that dissociation of HK2 from mitochondria promotes G6P channeling to glycogen synthesis and PPP, instead of using it as a fuel to mitochondrial metabolism [36].

**Figure 4:**
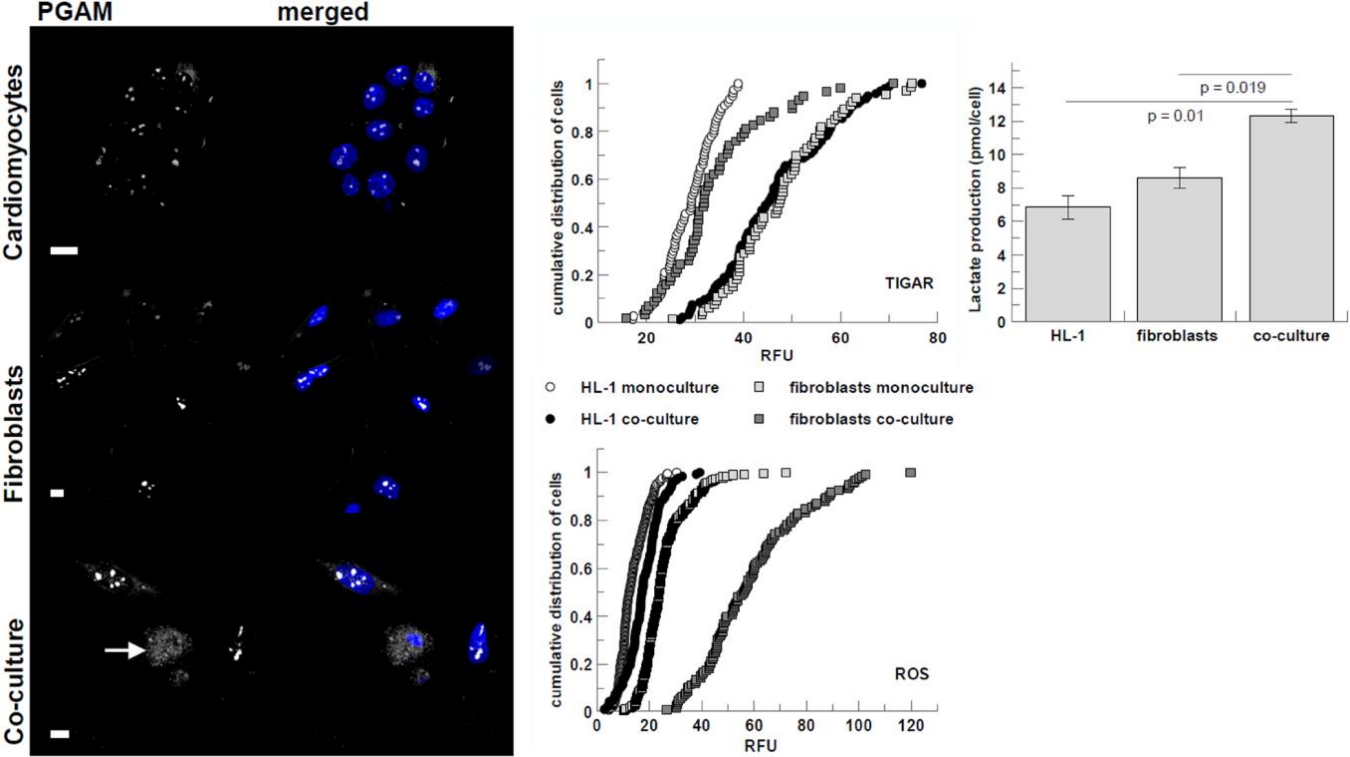
**Co-culturing-induced changes in visibility of PGAM2 C-terminus, immunostaining intensity of TIGAR, and ROS and lactate production.** PGAM2 is presented in white, nuclei are blue. White arrow point to cardiomyocytes. Bar = 10 μm. Lactate production is presented as pmol of lactate per cell per 48 h. Error bars represent standard deviation from three measurements. RFU – relative fluorescence units.

Although co-culturing apparently did not induce obvious changes in PFKM amount and distribution (data not shown), we observed opposing alterations in TIGAR expression in the two types of co-cultured cells: its reduction in fibroblast (D=0.6, p<0.001) and increase in HL-1 cells (D=0.76, p<0.001) (Figure 4). The TIGAR protein by lowering intracellular level of fructose-2,6-bisphosphate – a PFK activator, negatively regulates glycolysis and channelizes the intermediates to pentose phosphate pathway (PPP) [37] which generates putatively protective and reparative molecules. Inhibition of glycolysis in HL-1 cell supplied with an energetic substrate (lactate) by fibroblasts is compatible with the observations suggesting fibroblast-to-cardiomyocyte lactate shuttle. Additionally, the elevated expression of TIGAR can modulate the HL-1 cells apoptotic response to mild stress induced by augmented concentration of lactate excreted into the culture medium by fibroblasts.

Also in line with the hypothesis, in co-cultures, the C-terminus of PGAM2 became detectable in cytoplasm of cardiomyocytes (Figure 4). In standard monoculture conditions, this region of PGAM is usually exposed to solvent only in nucleoli (Figure 4; [38]). In cytoplasm, the C-terminal region of PGAM is involved in stabilization of the multienzymatic glycolytic complex (and thus, unavailable to antibodies) regulating the cellular rate of ATP synthesis in glycolysis. Disruption of this complex in a cell, induced for example by high concentrations of exogenous lactate, results in decrease of glucose oxidation to lactate, and correlates with emergence of the C-terminus of PGAM in cytoplasm [39].

We did not observe any noticeable changes in distribution and expression of aldolase in the co-cultures (data not shown). However, aldolase is one of the most abundant proteins in every cell type and its total concentration is not supposed to restrict the rate of glycolysis [4]. On the other hand, aldolase, together with PGAM2 and other enzymes of triose phosphate metabolism, is a part of the macromolecular complex crucial for the ATP synthesis in glycolysis [40]. We can deduce dissociation of such complex, and thus, reduction of glycolytic flux, in the co-cultured cardiomyocytes from the observation of the C-terminal region of PGAM2 in cytosol of these cells.

The PKM staining in the monocultures revealed that the enzyme is localized on mitochondria. In turn, in the co-cultures, PKM was densely packed around nuclei of both cell types (Figure 3). It has been shown that in cancer cells, PKM2 but not PKM1 promotes mitochondrial elongation by effecting the fusion (mitofusin 2) and fission (Drp1) proteins. The observed “energetic” outcomes of the fusion have been contradictory [41, 42] and necessitate further studies. However, to our knowledge, this is the first paper showing that PKM interact with mitochondria in healthy cells. Since the co-culturing induced similar changes both in fibroblasts and in cardiomyocytes, it is particularly hard to infer the metabolic meaning of this interaction.

Intriguingly, we found that co-culture resulted in considerable increase in ROS production in fibroblasts (D=0.79, p<0.001) (Figure 4). The ROS increase in fibroblasts is consistent with the observed reduction of TIGAR-related fluorescence in these cells – expression of TIGAR protein has been shown to be inversely related to intracellular ROS levels (see in [43]. Furthermore, it has been shown that mild oxidative stress can stimulate glucose uptake by up-regulating GLUT1 expression [44, 45], which we observed in fibroblasts from co-cultures (Figure 2). ROS can also up-regulate HIF-1A transcription and translation by several pathways (for review see in [46]).

An additional indicator of the increased glycolytic activity and lactate secretion by fibroblasts in the co-culture was the higher lactate accumulation in the medium collected from the co-culture in comparison with that detected in the monoculture media (Figure 4).

Together, it appears that co-culturing of cardiomyocytes with fibroblasts leads to orchestrated changes in protein expression/localization concurring with the fibroblasts-to-cardiomyocytes lactate shuttle hypothesis.

Careful screening of the co-culture plates revealed that these changes were independent on physical contacts between the two cell types (Supplementary Figure 1D). It has been shown, however, that the protein and nucleic acid cargo of extracellular vesicles, called microvesicles or exosomes [47], shed from fibroblasts mediates cross-talk between them and other cell types, e.g. cancer cells (for review see [48]). In response to stress, cardiac fibroblast–derived vesicles containing microRNA can mediate HL-1 cardiomyocyte hypertrophy [49]. In turn, the “cardiosomes” can transfer genetic information to fibroblasts [50]. Thus, we tested if the observed differences in protein localization/expression between the cells in mono- and co-culture emerged from paracrine signaling factors or vesicles secreted to the culture medium.

The culture medium from the cardiomyocyte-fibroblast co-culture was “fractionated” into supernatant and microvesicles fraction using ultracentrifugation. Then, the microvesicles were stained with a green fluorescent dye PKH67 and added to monocultures of fibroblasts and cardiomyocytes. Alternatively, the fibroblast and cardiomyocyte monocultures were treated with the unstained microvesicles or supernatant fractions and subcellular localizations of HIF1α, FBP2 and C-terminal region of PGAM2 were determined.

**Figure 5:**
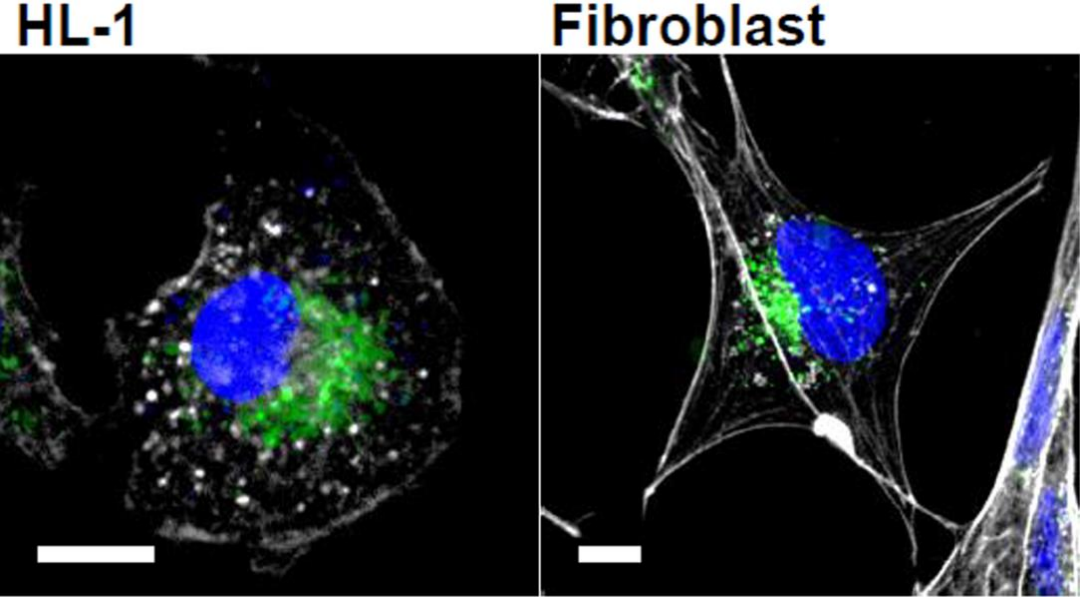
**Microvesicles uptake into cardiomyocytes and fibroblasts.** Cells were incubated with isolated and PKH67-labeled microvesicles (green) for 24 h. Actin is shown in white and nuclei in blue. Bar = 10 μm.

24h incubation of cells with PKH67-stained microvesicles resulted in their accumulation in both cell types (Figure 5), as it has been shown before [49].

**Figure 6:**
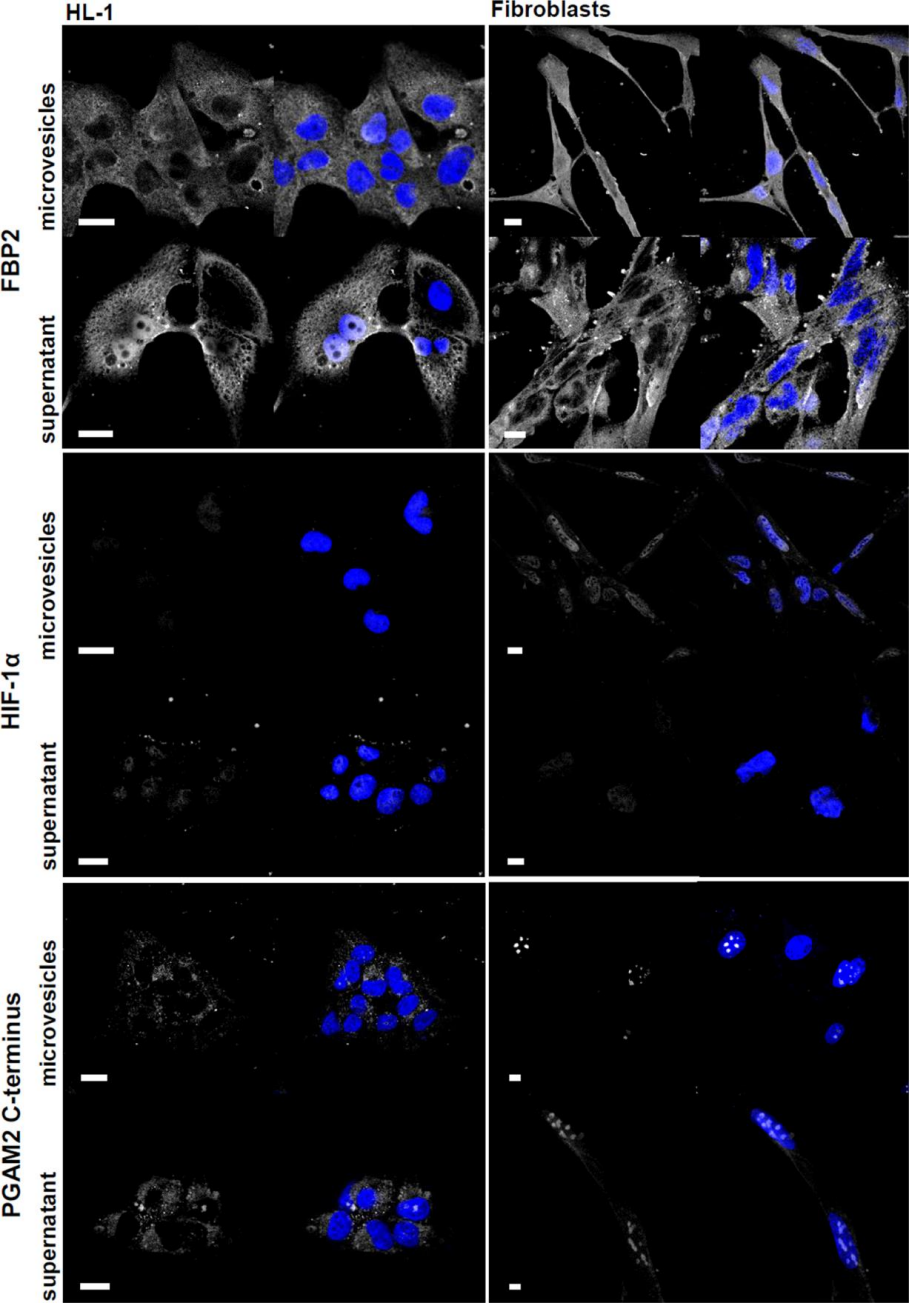
**Microvesicle- and supernatant-induced changes in cardiomyocytes and fibroblasts.** The studied proteins are shown in white, nuclei – in blue. Bar = 10 μm.

Treatment of the cells with isolated microvesicles mimicked the effect of the cardiomyocyte-fibroblast co-culture on FBP2 and HIF1α nuclear amount. In almost 70% of cardiomyocytes FBP2 was absent from the nucleus. In the same time, the enzyme accumulated in nuclei of about 80% of fibroblasts, and the HIF1α-related fluorescence increased markedly in nucleus of almost every cell. Depletion of microvesicles from the co-culture medium completely abolished these changes (Figure 6), which implies that a microvesicle-derived factor was responsible for the observed behavior of FBP2 and HIF1α.

By contrast, both microvesicles and supernatant fraction were able to induce the emergence of the C-terminus of PGAM in cytoplasm (Figure 6). This indicated, not unexpectedly, that the stability of glycolytic complexes was controlled by more than one factor.

Quite recently, based on the observed similarities in brain and heart glycolytic profiles, and in physiological response of these organs to hypoxia we have suggested that the classical cell-to-cell lactate shuttle operates not only between astrocytes and neurons but also between fibroblasts and cardiomyocytes [4, 6]. During the last decade various aspects of functional coupling between fibroblasts/cardiac epithelial cells and cardiomyocytes have been intensively studied. However, as yet, the effect of the cell-to-cell cross-talk on the expression of basic energy metabolism proteins is poorly understood. Some recent studies have demonstrated that cardiomyocytes may stimulate glycolytic flux in endothelial cells by releasing exosomes loaded with GLUT transporters and glycolytic enzymes [51]. This phenomenon is hypothesized to play an important role in the response of heart to hypoglycemic conditions [52]. By contrast, much less is known about the impact of cardiomyocytes-cardiac fibroblasts cross-talk on glycolysis. In this report, we demonstrated that co-culturing of these two types of cells changed expression and subcellular localization of proteins involved in regulation of glycolysis and lactate release/uptake (HIF-1α, FBP2, TIGAR, HK2, PKM2, PGAM2, GLUT-1, LDHA and B), in fibroblasts and cardiomyocytes, in a manner suggesting stimulation of glycolytic metabolism in fibroblasts and reduction of glycolytic capacity in cardiomyocytes. This coupling was mediated mainly by the cargo of microvesicles. However, the stability of glycolytic complex was regulated also by some soluble factor(s) released directly into the culture medium. This was not unexpected, as we have shown that such complex is regulated by concentration of metabolites, e.g. lactate [39]. Along with the changes in metabolic profiles, the co-culturing affected the proliferative potential of both cell types which was associated with changes in subcellular localization and/or phosphorylation status of proteins known to regulate both energy metabolism and cell cycle progression. The co-culture-induced changes resembled the postnatal switching from the “fetal” to “mature” cardiac phenotype. Such “switching” might be additionally substantiated by reduction of the “embryonic” smooth muscle actin [53] immunostaining intensity (from faint to virtually none) of both types of the co-cultured cells (data not shown).

Recently, we have shown that in aging hippocampi, there is a rise in production of extracellular matrix (ECM) proteins which can lead to spatial separation of astrocytes and neurons and impede metabolic cross-talk between them. In the same time, concentrations of glycolytic enzymes increase in neurons and, as a result, they become independent of astrocytic metabolism and stop consuming astrocyte-derived lactate. [54]. In aging heart, pathological hypertrophy accompanied by hypoxia and increase in ECM deposition is an independent risk factor for heart failure (for review see [55]). Furthermore, high glucose/diabetic conditions may stimulate production of ECM in heart [56].

The results described here show that the similarities of heart and brain proteomes demonstrated earlier extend to physiological level, and provide yet another piece of evidence that cardiomyocytes and fibroblasts form a metabolic syncytium in which the exchange of important chemical signals takes place not only through gap junctions but also through excretion of signals/vesicles into cellular milieu. This cross-talk induces transitions that mimic the postnatal shift in mode of cardiac growth. On the other hand, the similarity of aging-related changes in brain and heart (e.g. increase in ECM deposition) might suggest that the metabolic cross-talk between fibroblasts and cardiomyocytes is impaired in old animals and also in animals suffering from obesity-related myocardial insulin resistance.

In conclusion, these results imply that alterations in metabolic cooperation between fibroblasts and cardiomyocytes should be taken into account in course of designing therapeutic strategies for treatment of early-life cardiomyopathies that result from disruption of the maturation of cardiac metabolic pathways, and also heart failure associated with aging and metabolic complications, e.g. obesity-related diabetic conditions linked to ECM deposition and adversely affecting myocardial mechanical function and tolerance to ischemia and reperfusion.

## MATERIALS AND METHODS

### Cell lines and chemicals

HL-1 cardiomyocytes, a murine cell line [57] was a gift from Dr. W.C. Claycomb (Louisiana State University Health Science Center, New Orleans, LA, USA).

Murine cardiac fibroblasts were isolated from hearts of newborn C57BL/6 mice according to [58]. The protocol of isolation was approved by the II Local Scientific Research Ethical Committee, Wroclaw University of Environmental and Life Sciences and every effort was made to minimize the number of animals used for the experiments.

If not stated otherwise, all the chemicals were bought from Sigma-Aldrich.

### Cell culture

HL-1 cardiomyocytes were cultured in Claycomb medium as described before [13]. Fibroblasts were initially cultured in glutamine-containing DMEM low glucose medium supplemented with 10% fetal bovine serum and penicillin (100 units/ml)/streptomycin (100 mg/ml). The cells were maintained at 37°C under 5% CO_2_. To test the effects of co-culturing, the cardiomyocytes and fibroblasts were seeded together (in the 3 to 1 ratio [59]) or alone on coverslips covered with fibronectin, and cultured in Claycomb medium for 48 h.

### Immunocytochemistry

Cells growing on coverslips were fixed in 4% paraformaldehyde, permeabilized with 0.1% Triton X-100 in PBS and incubated with 3% BSA in PBS to reduce nonspecific binding of antibodies. Then cells were incubated overnight with a primary antibody and 1 h with an appropriate secondary antibody (Supplementary Table 1). To visualize nuclei, actin cytoskeleton and mitochondria, the cells were counterstained with respectively, DAPI, Phalloidin-Alexa 488 (ThermoFisher) and MitoTracker Deep Red (ThermoFisher). To avoid confusion resulting from the use of secondary antibodies conjugated to different fluorochromes, the studied proteins are always presented in pictures as white.

Images were acquired on FV-1000 confocal microscope (Olympus) with 60x (oil, Plan SApo, NA=1.35) objective using the Sequential scan option. The fluorescence intensity was measured and the corrected total cell fluorescence (CTCF) of individual cells was calculated using the Cell^F software (Olympus).

If the changes in fluorescence subcellular distribution were not evident in pictures, data was presented in percentage frequency (“cumulative distribution”) plots (created with GraFit program). For statistical analyses of cumulative distribution plots the two sample Kolmogorov–Smirnov test was used. The experiments were repeated three times with similar results and representative data from one experiment is shown in figures.

### Cell cycle analysis

Cell cycle analysis was performed using a Muse™ cell cycle kit from Millipore according to the manufacturer’s instructions. Briefly, cells were seeded in the same density as mono- and co-cultures. After 48 h they were harvested by trypsinization, washed with PBS and fixed with 70% ethanol (-20°C, 5 h). Then they were incubated with 200 μl of the Muse Cell Cycle Reagent for 30 min at room temperature in the dark. The percentage of cells in G0/G1, S and G2/M phases was then calculated using a Muse Cell Analyzer (Millipore).

### Microvesicle purification and labeling

After 48 h of cardiomyocyte-fibroblast co-culture, the culture medium from 75 ml culture flask was collected and microvesicles were purified according to the method described in [50]. To confirm their isolation and uptake by cells, microvesicles were stained with PKH67 Green Fluorescent Cell Linker Kit and incubated for 24 h (as described in [49]) with cardiomyocytes and fibroblasts monoculture. Then, the cells were fixed in 2% paraformaldehyde, counterstained with phalloidin-Alexa 633 (ThermoFisher) and DAPI and observed in confocal microscope. Alternatively, the monocultures were treated for 48 h with unstained microvesicles or supernatant remained after the isolation procedure and subcellular localization of HIF1α, FBP2 and C-terminal region of PGAM2 was determined.

### Measurement of cellular ROS production

To measure intracellular ROS production, the cells growing on coverslips were loaded with 5 μM dihydrofluorescein diacetate (H2DCF-AC; 20 min, 37°C), thoroughly rinsed with Hank’s Balanced Salt Solution, and mounted on slides. Live cells were examined with the Olympus IX71 fluorescence microscope equipped with the Cell^F software (Olympus). The fluorescence of the dye was excited at 488 nm for 500 ms. the corrected total cell fluorescence (CTCF) of individual cells was calculated using the Cell^F software (Olympus), and presented in percentage frequency (“cumulative distribution”) graph. The experiments were repeated three times with similar results and representative data from one experiment is shown in Figure 4. Collected data was analyzed as described above (“Immunocytochemistry”).

### Lactate assay

Lactate release into culture medium was assayed based on spectrophotometric measurement of NAD+ reduction at 340 nm (as described in [60]), using samples of the medium from the mono- and co-cultures after 48 h of the culture. The measurement of NAD+ reduction monitored at 340 nm was performed with the Agilent 8452A diode array spectrophotometer. The experiment was performed in triplicate. The results are expressed as mean and standard deviation. For an evaluation of statistical significance the Student’s t-test was used. A probability of p < 0.05 was considered to represent a significant difference.

## Supplementary Material

Supplementary Figure 1

Supplementary Table 1
