# Supplementary material for: Cell-to-cell lactate shuttle operates in heart and is important in age-related heart failure
Source: Aging (Albany NY). 2020 Feb 8;12(4):3388–406. doi: 10.18632/aging.102818 (PMC7066931; doi:10.18632/aging.102818)
Supplement: Supplementary Figure 1 [file aging-12-102818-s002..pdf]

SUPPLEMENTARY FIGURE

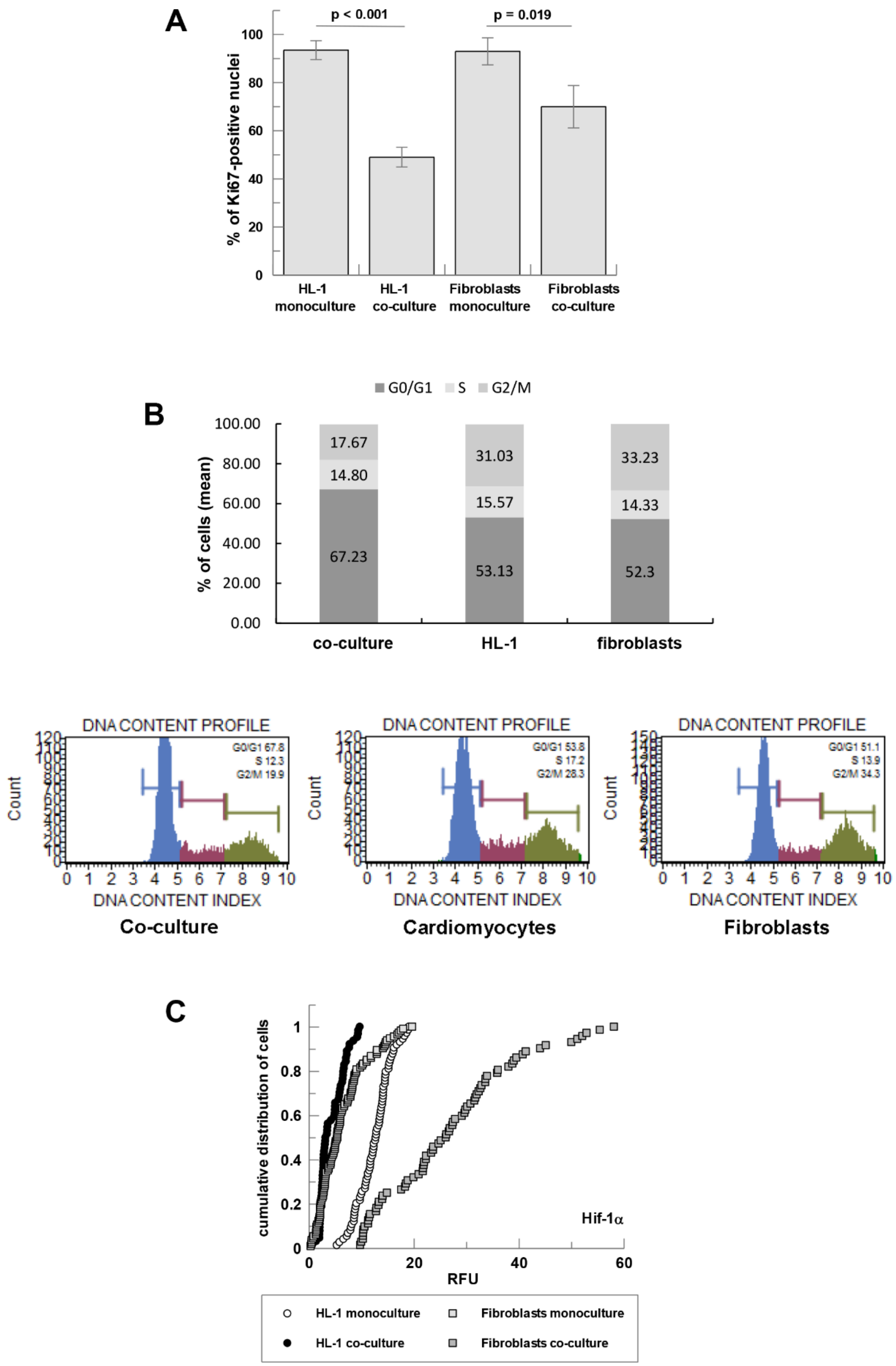

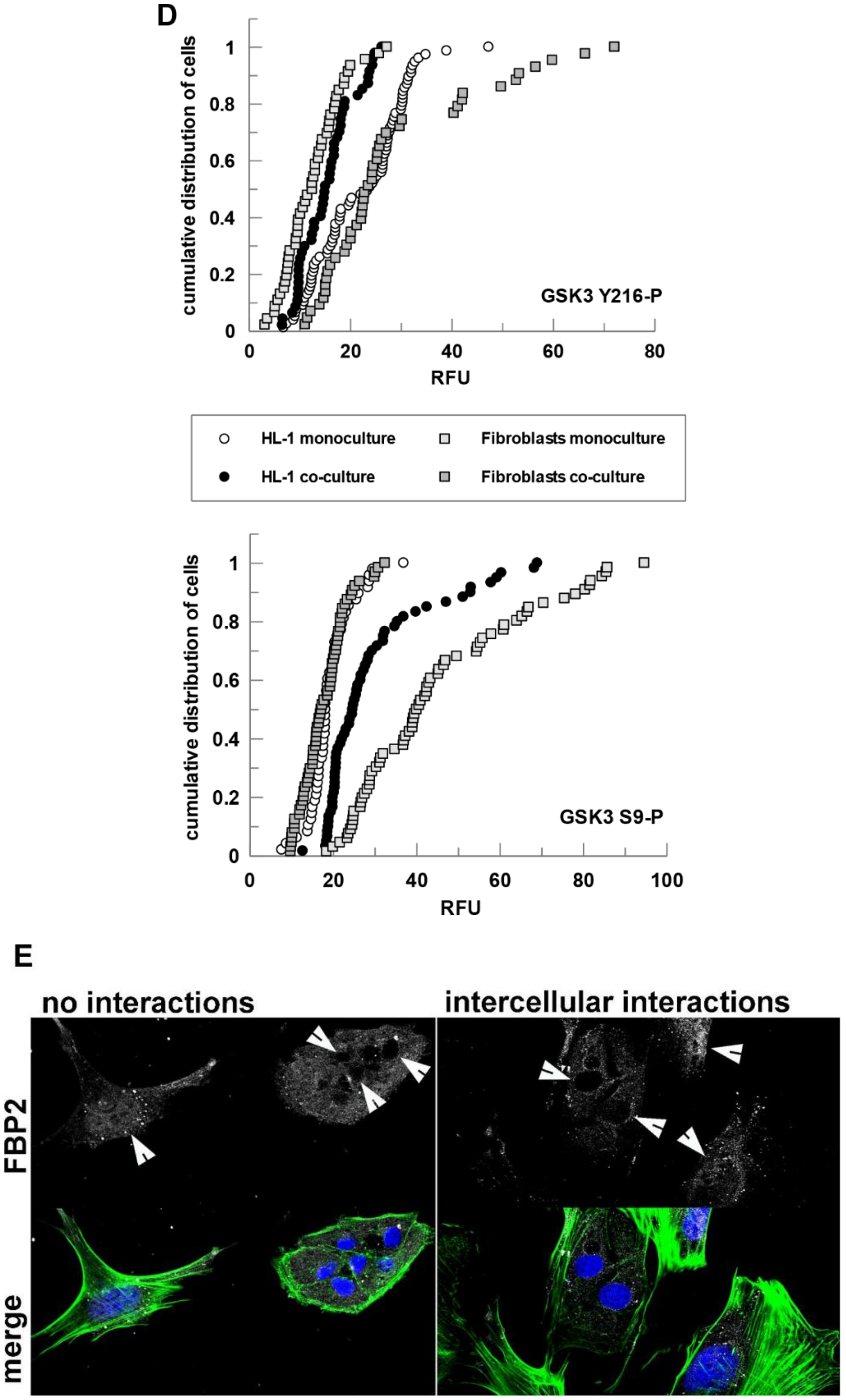

Supplementary Figure 1. Changes in protein localization/expression and cell cycle progression induced by co-culture of cardiomyocytes and fibroblasts. (**A**) Number of Ki67-positive nuclei in cardiomyocytes and fibroblasts, in mono- and co-cultures. The results are expressed as mean and standard deviation. For an evaluation of statistical significance, the Student's t-test was used. (**B**) Cell cycle progression in the co-culture of cardiomyocytes and fibroblasts compared to the monocultures. The graph presents mean percentage (from 3 independent experiments) of the cells in a given phase of the cell cycle. Representative histograms from one experiment are also shown. (**C**) Changes in Hif-1α-related fluorescence in fibroblasts cultured alone and with HL-1 cells. The percentage frequency ("cumulative distribution") plot was created with GraFit program. For statistical analysis the two sample Kolmogorov–Smirnov test was used. RFU – Relative Fluorescence Units. A noticeable increase of anti-HIF-1α staining was observed in nuclei fibroblasts co-cultured with HL-1 cells, as compared monoculture (D=0.81, $p<0.001$). In HL-1 cells, co-culturing with fibroblasts induced decrease of HIF-1α-related fluorescence in nuclei (D=0.85,

p<0.001). (**D**) Changes in fluorescence of the active (Y216-P) and inactive (S9-9) form of GSK3 in nuclei cells, in mono- and co-cultures. The percentage frequency ("cumulative distribution") plot was created with GraFit program. For statistical analysis the two sample Kolmogorov–Smirnov test was used. RFU – Relative Fluorescence Units. A decrease of Y216-P (D=0.45, p<0.001) and increase of S9-P (D=0.53, p<0.001) was observed in cardiomyocytic nuclei in co-cultures, compared to monocultures. In turn, in the fibroblasts' nuclei, the amount of Y216-P increased (D=0.64, p<0.001) and S9-P decreased (D=0.8, p<0.001) in co-cultures. (**E**) Changes in the studied proteins localization/expression were independent on physical contacts between the two cell types. During the course of the study we tested different densities of HL-1–fibroblasts co-cultures and obtained the same results. Below, FBP2 (white) disappears from HL-1 cells nuclei and accumulates in nuclei of fibroblasts in the co-cultures, regardless of intercellular contacts. Actin is counterstained green (phalloidin-Alexa 488), nuclei – blue (DAPI). Arrows point to some nuclei.
