# Supplementary material for: Cell-to-cell lactate shuttle operates in heart and is important in age-related heart failure
Source: Aging (Albany NY). 2020 Feb 8;12(4):3388–406. doi: 10.18632/aging.102818 (PMC7066931; doi:10.18632/aging.102818)
Supplement: Supplementary Table 1 [file aging-12-102818-s001..pdf]

SUPPLEMENTARY TABLE

Supplementary Table 1. The list of antibodies used in the experiments.

Antigen	Animal host	Catalog number	Vendor/Reference	Dilution
**Primary antibodies**				
Fructose 1,6-bisphosphatase (FBP)	rabbit	-	[1]	1:800
C-terminal peptide of phosphoglucomutase 2 (PGAM2)	goat	NB100-774	Novus Biologicals	1:1000
Aldolase A	rabbit	-	[2]	1:300
Hexokinase 2	rabbit	AB3279	Sigma-Aldrich	1:400
Glycogen synthase kinase-3 beta (GSK3, total protein)	mouse	610201	BD Biosciences	1:300
GSK3 phospho-Ser9	rabbit	04-1075	Merck	1:250
GSK3 phospho-Tyr216	mouse	612612	BD Biosciences	1:150
Hypoxia-inducible factor 1-alpha (HIF-1 alpha)	rabbit	bs-0737R	Bioss	1:500
Proliferation marker protein Ki67	rabbit	301107	Novocastra	1:100
Phosphofructokinase muscle form (PFKM)	rabbit	HPA002117	Atlas Antibodies	1:200
Pyruvate kinase muscle form (PKM)	goat	R1108	Acris	1:400
Lactate dehydrogenase A (LDHA)	rabbit	NBP1-48336	Novus Biologicals	1:200
Lactate dehydrogenase B (LDHB)	rabbit	ab75167	Abcam	1:400
Monocarboxylate transporter 1 MCT1 (SLC16A1)	mouse	ab90582	Abcam	1:100
Monocarboxylate transporter 2 MCT2 (SLC16A7)	rabbit	bs-3995R	Bioss	1:100
Monocarboxylate transporter 4 MCT4 (SLC16A3)	rabbit	HPA021451	Atlas Antibodies	1:50
TP53-induced glycolysis and apoptosis regulator (TIGAR)	rabbit	PRS4051	Sigma-Aldrich	1:400
Glucose transporter 1 (GLUT1)	mouse	ab40084	Abcam	1:50
**Secondary antibodies**				
anti-rabbit Alexa 633-conjugated	goat	A21070	ThermoFisher	1:400
anti-goat FITC-conjugated	rabbit	F9012	Sigma-Aldrich	1:200
anti-mouse TRITC-conjugated	goat	T6528	Sigma-Aldrich	1:200

1. Gizak A, Dzugaj A. FBPase is in the nuclei of cardiomyocytes. FEBS Lett. 2003; 539:51–5. https://doi.org/10.1016/s0014-5793(03)00183-2; PMID: 12650925

2. Mamczur P, Dzugaj A. Nuclear localization of aldolase A in pig cardiomyocytes. Histol Histopathol. 2004; 19:753–8. https://doi.org/10.14670/HH-19.753. PMID: 15168337
